# Sensorimotor Uncertainty of Immersive Virtual Reality Environments for People in Pain: Scoping Review

**DOI:** 10.3390/brainsci13101461

**Published:** 2023-10-16

**Authors:** Mar Flores-Cortes, Javier Guerra-Armas, Consolacion Pineda-Galan, Roy La Touche, Alejandro Luque-Suarez

**Affiliations:** 1 Faculty of Health Sciences, University of Malaga, 29071 Malaga, Spain; 2 Instituto de Dolor Craneofacial y Neuromusculoesquelético (INDCRAN), 28008 Madrid, Spain; 3 Departamento de Fisioterapia, Centro Superior de Estudios Universitarios La Salle, Universidad Autónoma de Madrid, 28023 Madrid, Spain; 4 Motion in Brains Research Group, Institute of Neuroscience and Sciences of the Movement (INCIMOV), Centro Superior de Estudios Universitarios La Salle, Universidad Autónoma de Madrid, 28023 Madrid, Spain; 5 Instituto de Investigacion Biomedica de Malaga (IBIMA), 29071 Malaga, Spain

**Keywords:** sensorimotor, uncertainty, virtual reality, motor behaviour, pain

## Abstract

Introduction: Decision making and action execution both rely on sensory information, and their primary objective is to minimise uncertainty. Virtual reality (VR) introduces uncertainty due to the imprecision of perceptual information. The concept of “sensorimotor uncertainty” is a pivotal element in the interplay between perception and action within the VR environment. The role of immersive VR in the four stages of motor behaviour decision making in people with pain has been previously discussed. These four processing levels are the basis to understand the uncertainty that a patient experiences when using VR: sensory information, current state, transition rules, and the outcome obtained. Methods: This review examines the different types of uncertainty that a patient may experience when they are immersed in a virtual reality environment in a context of pain. Randomised clinical trials, a secondary analysis of randomised clinical trials, and pilot randomised clinical trials related to the scope of Sensorimotor Uncertainty in Immersive Virtual Reality were included after searching. Results: Fifty studies were included in this review. They were divided into four categories regarding the type of uncertainty the intervention created and the stage of the decision-making model. Conclusions: Immersive virtual reality makes it possible to alter sensorimotor uncertainty, but studies of higher methodological quality are needed on this topic, as well as an exploration into the patient profile for pain management using immersive VR.

## 1. Introduction

### 1.1. Pain Conceptualisation and Its Relationship with the Environment

Pain has been conceptualised as a disturbance in the interactive relationship between the subject and the world [1]. It is part of a motivational system that urges the individual to take action when the integrity of the body is challenged [2]. When pain and suffering persist, they become embodied as a part of the person [3] and may deeply affect different aspects of the person’s life. Even the sense of inhabiting the world can be profoundly altered [4] as people experiencing pain are often no longer able to flexibly attune to the environment in the way they were before [5]. Even though the updated information available from the internal and external environments is regularly used to estimate the risk of threats, this process is full of uncertainties [6].

### 1.2. Dynamics of Perception, Action, and Stress Responses

Sensory data from the world and the body are necessary to act through behavioural and stress responses. Both perceptions and actions aim to minimise prediction errors (uncertainty) [7]. 

When we feel threatened by changes in the external or internal environment, we are confronted with the question ‘What strategy should I select to safeguard my future physical, mental, and social wellbeing? That is when “stress” arises, as we are uncertain about the possible answer–reaction, with a lack of control potentially appearing [7]. Stressful situations—from a bio-psychological perspective—have been characterised by ‘no information, no control, and uncertainty with a sense of threat’ [8].

During action and perception tasks, subjects behave in order to minimise threats and the negative consequences of uncertainty [9]. Uncertainty about a variable means that we do not know its true state or magnitude, as the variable can express one of several possible values [10] and it biases our decision making [11]. When a person feels uncertainty and threat, they enter into a hypervigilant state to decrease uncertainty (about strategy selection) as fast as possible [12]. This is because of a changing internal or external environment. During a movement, the nervous system blends noisy sensory signals with noisy output signals from motor commands. This integration serves the purpose of estimating the body’s state and this mechanism aids in reducing uncertainty regarding whether the sensory information is a result of one’s own actions or external events [13]. Most of our daily activities have a time constraint for successful completion and involve asynchronous processing of noisy sensory information and the generation of actions with uncertain outcomes [14]. This results in a competition between the time allocated to sensing and the time spent on acting, described in two earlier studies as a sensorimotor trade-off [9,15]. Sensory information regarding an object’s location can be affected by disturbances, leading to a lack of precision in perceiving the object’s position. Similarly, motor commands may introduce inaccuracies and variations in movements [13].

### 1.3. Precision, Uncertainty, and Sensorimotor Behaviour

**Figure 1:**
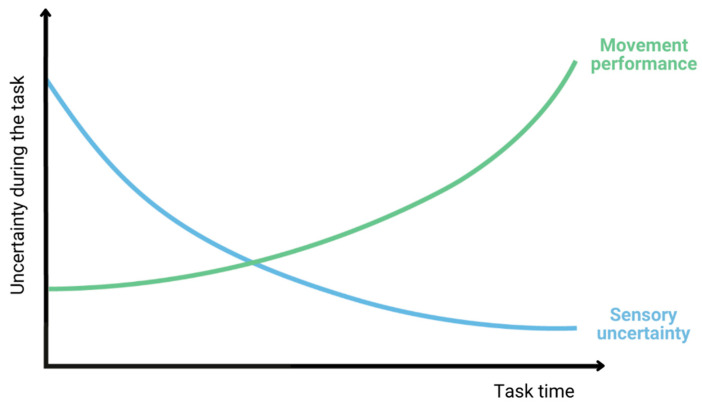
During the time of performing a task, sensory uncertainty decreases with time, while movement performance increases. The combined sensorimotor uncertainty, which is the sum of the sensory and motor uncertainty, is shaped like a valley and has a minimum. It starts with the decrease in sensory uncertainty (blue), and from the point of the intersection of the lines, it increases through movement performance (green).

Successful behaviour requires a combination of sensation and action across time [15]. The degree to which sensory feedback is integrated into an ongoing movement and the degree to which movement errors drive adaptive changes in feedforward motor plans scales inversely with sensory uncertainty [16]. The process of executing goal-directed movements involves multiple different stages. Initially, it is necessary to pinpoint both the target and the leg’s location. Subsequently, motor commands must be formulated to guide the leg to the desired target location. Finally, these motor commands are transmitted to the arm muscles, resulting in the actual movement. Throughout these stages, neural noise contributes to uncertainty [13]. Essentially, sensory uncertainty decreases with time, while motor uncertainty increases with time (Figure 1). The combined sensorimotor uncertainty, which is the sum of the sensory and motor uncertainty, is shaped like a valley and has a minimum value [17].

The totality of the human experience is complex and, according to the enactive model [4], it is intrinsically embodied and embedded in an environment [5]. Noise and uncertainty are inherent to complex systems [18]. Real world behaviour requires the combination of a stream of sensory information and motor actions over time, where both sensory inputs and motor outputs are subjected to uncertainty [14]. Optimal motor planning takes into account uncertainty in sensory information [10]. Two parallel systems seem to intertwine in the motor cortex to create an integrated–isolated pattern: effector-specific regions (foot, hand, mouth) for isolating fine motor control and a mind–body interface (MBI) for the integrative whole-organism coordination of goals, physiology, and body movement [19].

### 1.4. Virtual Reality and Its Role in Pain Perception

**Figure 2:**
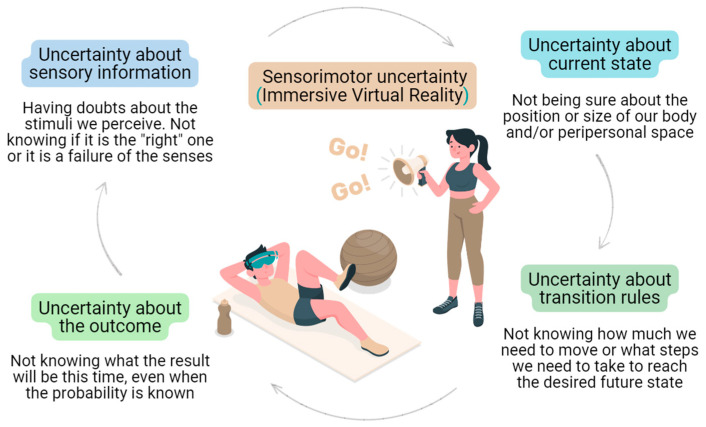
The four processing levels in motor behaviour decision making and the types of uncertainty a patient can face when immersed in a virtual reality environment in a context of pain. Uncertainty about sensory information: having doubts about the stimuli we perceive; not knowing if it is the right one or is a failure of the senses. It can be inherent to the environment or due to internal noise. Uncertainty about current state: not being sure about the position or size of our body and/or peripersonal space. Uncertainty about transition rules: not knowing how much we need to move or what steps we need to take from our current state to reach the desired future state; not knowing what outcome a change in behaviour leads to. Uncertainty about the outcome: not knowing what the result will be this time, even when the probability is known.

Motor behaviour is strongly influenced by sensorial uncertainty and the expected consequences of actions [20]. Virtual reality (VR) differs in both aspects from natural environments. Perceptual information in VR is less reliable than in natural environments as more noise is presented [21]. In the initial stages of the movement, when the current state estimate is deemed to be accurate, the predicted position from the forward model carries significant weight. As the movement progresses, and the estimate becomes less dependable, there is a gradual transition in weighting towards the feedback process [13]. Sensorimotor incongruity in immersive virtual reality environments or avatars can enhance uncertainty and can affect the capacity to integrate diverse sensory stimuli. This phenomenon may be particularly altered in people in pain, as summarised in a recent review by Vitterso et al. [22]. The role of immersive VR in the four stages of motor behaviour decision making in people with pain has been previously discussed [23]. These four processing levels can be useful to review the types of uncertainty that a patient can face when using VR in a context of pain (Figure 2).

To execute a goal-directed reaching movement, the nervous system must initially acquire spatial details concerning both the target and the leg. These details encompass not only their positions but also factors like orientation, size, and shape. Sensory input plays a crucial role in estimating these parameters. However, it is important to note that sensory signals have limitations in the amount of information they convey about both the external environment and the body’s state. At the neural level, these limitations manifest as neural noise, which gives rise to imperfections in precision (referring to variable errors and uncertainty) [13].

Precision is inherently constrained by spatial and geometric factors and the characteristics of sensory receptors. In the visual system, precision is contingent upon both position and direction. For a given position, the precision varies depending on the direction under consideration. Visual localisation becomes less precise as the distance from the observer increases, and depth perception is typically less accurate than determining the horizontal direction (azimuth). This discrepancy underscores the challenge that the visual system faces in estimating any distance compared to direction [24].

Conversely, when it comes to proprioceptive localisation, precision diminishes as the distance from the shoulder increases. Interestingly, localisation is more accurate in the depth perception than in the horizontal direction [25].

In the process of integration, all available information is combined in a manner that seeks to minimise the uncertainty within the overall estimate. This implies that the integration can only be comprehended when considering the impact of uncertainty.

Having information about the leg position is a crucial component in the planning of goal-directed arm movements. However, due to motor noise, the executed movements typically deviate from the intended ones. Nevertheless, it is remarkable that we often successfully reach the intended target despite these deviations. The calculation of the cost of various movements is unnecessary, as the optimal trajectory can be acquired through the accumulation of experience from repeated movements [13].

This suggests that, even during the execution of a movement, the estimation of the leg’s position remains crucial. However, during the progression of a movement, another source of information comes into play: an efference copy of the motor commands transmitted to the muscles. This efference copy serves the purpose of predicting the outcome of these motor commands [26].

Virtual reality (VR) refers to simulated experiences with multisensory content (visual, auditory, haptic, etc.), intentionally presented to the individual’s senses [27]. Nonetheless, there is a range of relatively varied and heterogeneous definitions of VR that can be found in the literature [28]. The specific features of virtual reality make the difference between all these types of varieties. Within these characteristics, three features are of particular relevance when presenting a situation of sensorimotor uncertainty within a VR environment: interactivity, immersion, and presence. 

Interactivity refers to the level of participation allowed by the user in the virtual reality environment. Interactive virtual reality allows users to navigate within the virtual space and interact with virtual objects and avatars [29]. 

Immersion is defined as an objective property of the system, to the extent to which a VR system can support natural sensorimotor contingencies for perception including the response to a perceptual action [30]. Presence is understood as the subjective experience of being in a place or environment, even when the person is physically in another place, with the user easily “forgetting” their presence in a computer-generated simulation [31]. 

Our central nervous system (CNS) has evolved to optimise motor behaviour by detecting sensory mismatches, which are continuously gathered and analysed to effectively navigate in a dynamically changing environment. Rapid and accurate detection of such discrepancies is critical for accurate interactions in virtual environments that can cause a loss of the sensation of immersion and presence. 

Higher levels of presence and higher levels of multisensory experience delivered within an immersive virtual environment are related to greater hypoalgesic effects compared to non-immersive VR [30,32]. Similarly, increased interactivity, i.e., interacting with virtual objects within an immersive environment, significantly increases the presence and is significantly related to a decrease in pain intensity compared to passive VR or no VR intervention [32]. These findings show the significance of the immersion and presence of the individual with pain within the immersive environment, and how sensory uncertainty may have an impact on the hypoalgesic effects produced by VR. 

The most commonly studied mechanism of VR has been distraction (78.6%) followed by embodiment (17.1%). However, distraction appeared to be the mechanism used in the majority of acute pain studies (97.8%), while embodiment was more common in chronic pain (54.5%) [33]. 

Distraction refers to the redirection of an individual’s attentional resources away from their pain, towards other stimuli (visual, auditory, tactile, and cognitive). It therefore “reduces” the cognitive ability to process pain. Virtual reality distraction has been used effectively to reduce acute pain. However, its use provides short-term effects when pain is persistent [34,35]. This can be explained as more than one mechanism can be present in the hypoalgesic effects of immersive VR [23,35].

Thus, the hypoalgesic effects of VR are the result of a competition for the limited attentional resources shared between the sensory inputs proposed by VR and the incoming nociceptive signals [36]. The reduction in pain with VR corresponds with changes in analgesic brain activity in areas associated with attentional processes, which are more active during distraction [37].

Findings reported by Limanowski [38] suggest that endogenous attention can balance the visual versus proprioceptive stimulus gain by contextualising their influence on multisensory areas representing the body for action in VR experiments. This allows redirecting these attentional resources of our CNS towards the visual stimuli presented in immersive VR environments, and consequently, the generation of these hypoalgesic effects. 

The ability of an immersive virtual reality system to elicit a vivid interactive experience, where features associated with increased pain reduction but also increased motor learning are enhanced, is the key to optimising clinical outcomes in patients living with pain. Therefore, this review aims to present the situations in which a person with pain may encounter sensorimotor uncertainty within an immersive virtual reality environment and how to manage these events to obtain the expected results. Thus, the objective of the study is to explore the effects of sensorimotor incongruence in immersive virtual reality (IVR) environments on pain perception, with a specific focus on understanding the mechanisms of uncertainty that patients experience. This scoping review also evaluates the role of immersion, interactivity, and presence in eliciting hypoalgesic effects and the importance of sensory input in the decision-making stages of motor behaviour. Finally, the review aims to propose strategies to optimise clinical outcomes by managing the uncertainties faced by pain patients in IVR environments.

## 2. Methods

### 2.1. Study Design

A scoping review is “a form of knowledge synthesis, which incorporate a range of study designs to comprehensively summarise and synthesise evidence with the aim of informing practice, programs, and policy and providing direction to future research priorities”. This review followed PRISMA recommendations. The review was composed of five steps: (1) defining the research question; (2) identifying relevant studies; (3) selecting the studies; (4) charting the data; and (5) collating, summarizing, and reporting the results to inform practice and future research. This review was guided by the following research question: Which types of uncertainty can a patient face when immersed in a virtual reality environment in a context of pain?

### 2.2. Search

We developed a search strategy using MeSH terms and keywords (virtual reality, immersive virtual reality, uncertainty, incongruence, sensorimotor feedback, and pain). We searched PubMed, PEDro, Cochrane CENTRAL, and SPORTDiscus from inception up to July 2023. We searched the grey literature (Open Grey and Google Scholar) to identify relevant unpublished work. We also searched the reference lists of the included trials and journals related to the scope of our study. Only trials that were written in Spanish and/or English were included. There were no ethnicity, setting, and gender restrictions.

With respect to the eligibility criteria (Table 1), the selection criteria used in this review were based on studies whose primary aim was either to describe uncertainty or incongruence in immersive virtual reality environments and/or to discuss (without the need to evaluate the extent/effectiveness of) the impact of this uncertainty on the pain of people who are exposed to immersive virtual reality (e.g., hypoalgesic effect, immersive virtual reality conflict, correspondence with motor decision making stage, modifying the environment).

### 2.3. Selection of Articles

**Figure 3:**
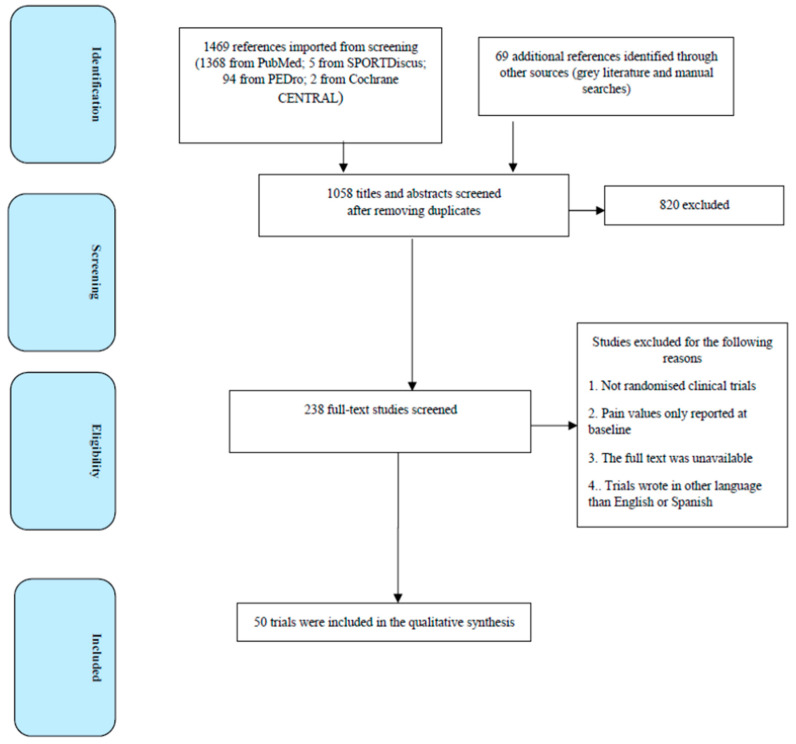
PRISMA flow diagram for study selection.

We screened potential articles by title and abstract after removing duplicates. We eliminated duplicates manually. Two reviewers independently performed the trial selection. If the trial selection was unclear after reading the title and abstract, we screened the full text. We resolved any disagreements via consensus or by a third reviewer if required. The trial selection process is shown in Figure 3.

### 2.4. Data Summary and Synthesis

Characteristics of included articles are summarised in Appendix A. We extracted the following information from each included trial: study year; stage of uncertainty; type of conflict generated by virtual reality; and main findings. We resolved any disagreements via consensus or by a third reviewer if required.

Regarding the results, the type of sensorimotor uncertainty, immersive virtual reality conflict, and correspondence with motor decision making stage regarding immersive VR interventions within the included studies were summarised narratively.

## 3. Results

Figure 3 provides a PRISMA (Preferred Reporting Items for Systematic Reviews and Meta-Analyses) flow diagram for the search process and study selection. A total of fifty articles were included in this scoping review.

Findings showing the relationship between sensorimotor uncertainty, motor decision making, and pain experience are found in Table 2.

### 3.1. Uncertainty about Sensory Information

Our system uses multisensory information to estimate surrounding features and to interact with objects. Visuotactile congruence has been studied in order to understand how our system is capable of estimating the weight of an object, within an illusion created by VRi [39]. This study shows that in the presence of sensorimotor conflict in combination with incongruent visuotactile stimuli, tactile cues have a stronger influence on the perceived heaviness than visual cues. Furthermore, the interaction with virtual objects in an immersive environment influences pain intensity [29]. These results show that interaction with objects increases the perception of presence, decreases pain intensity, and modulates threat perception compared to a passive virtual reality. 

When visuotactile incongruence occurs, it can affect the strength of the virtual avatar’s perception of body ownership [40]. Both spatial and temporal timing of tactile and visual stimuli can increase sensory uncertainty and can disrupt this bodily illusion. An asynchrony greater than 600 ms between stimuli is sufficient to affect multisensory integration within immersive virtual reality (VRi) [41]. Several studies have shown that enhancing tactile feedback by providing appropriate visuotactile congruency enhances the hypoalgesic effects of the VRi intervention [42,43].

The cingulate cortex and network of the parieto-occipital cortex may contribute to prediction errors when manipulating visuotactile congruency within a VRi setting [44]. However, cortical activity in the posterior parietal cortex and visual cortex associated with a prioritisation of vision over proprioception has been found when inducing an attentional setup. This occurs in those participants that prioritise one sensory modality over the other when a visuo-proprioceptive conflict is presented [38]. These neural signatures might be useful for detecting sensorimotor uncertainty in user predictions when interacting with virtual worlds. 

Similarly, the congruence between visual and auditory cues can decrease this uncertainty, with a positive effect on both the perception of the virtual environment [45] and the motor behaviour [46] within it. However, a visual-auditory incongruence may have an impact on the perceived location of a visual object and peripersonal space [47].

Several authors have discussed the relationship between visuo-vestibular incongruence and one of the most frequent adverse effects in the use of VRi, namely cybersickness [48,49]. When visual inputs are not correlated with vestibular information, as can occur when an immersive virtual environment is moving while the subject with the head-mounted device is not, the uncertainty increases due to conflicting sensory inputs [50]. This phenomenon has been associated with the speed at which the virtual environment moves can have an influence on the likelihood of causing cybersickness. Speeds from 3 m/s to 10 m/s progressively increase sickness symptoms [51].

### 3.2. Uncertainty about Current State

The congruence between visual information and proprioception seems to be crucial for the system’s perception of the body’s position in space, as well as for defining the peripersonal space (EPP). Research has explored the effect of experimentally inducing a visuo-proprioceptive incongruence between the virtual hand and the subject’s real hand. In the study conducted by Fossataro et al., greater visuo-proprioceptive uncertainty resulted in the system becoming more sensitive to identifying the boundaries of the peripersonal space and encoding the size of the hand as larger [52]. These results show the neuroplasticity of the central nervous system in shaping body ownership and the ability to cope with potential threats in the presence of uncertain sensory cues.

The studied relationship between body disownership, pain perception, and reduced top-down modulation by placebo [53], together with these findings, might offer a hypothesis on how to modulate body representation through VRi by increasing uncertainty about different sensory signals in people with pain. This hypothesis has been tested in the studies by Matamala-Gomez et al. [54,55], who found that body illusions induced by VRi can generate both a decrease and an increase in pain perception in subjects with chronic pain compared to healthy subjects. Modifying the visual appearance (size, colour and/or transparency), while inducing the illusory ownership over the virtual arm, generates a pain reduction response. Other studies have reported the same in healthy subjects where the modification of the visual appearance of the embodied avatar’s arm produced hypoalgesic effects on experimentally induced pain [56].

Thus, enhancing the uncertainty of the current state of the body’s representation through virtual avatars in an immersive environment may change the person’s pain experience.

### 3.3. Uncertainty about Transition Rules

Immersive virtual reality could modify the person’s relationship with their body and with the environment [23]. There is a relationship between the perception of presence in VRi and pain tolerance [57]. Greater multisensory congruence leads to a greater presence within the virtual world [30]. This modifies the threat perception and peripersonal space. A greater presence has been related to a decrease in the occurrence of cybersickness [48] and to an improvement in task performance [58].

Likewise, the relationship between the sense of agency of a virtual avatar, body representation, and peripersonal space has been studied [59]. In this experiment, the manipulation of the sense of agency on an external object within a virtual immersive environment induced changes in the body schema and the peripersonal space when an adequate visuomotor congruence was achieved between the virtual avatar and the real subject. However, when there was greater sensorimotor uncertainty, this response did not occur. It has been observed that when greater visuomotor uncertainty in the movement trajectory is added, the reported perception of ownership and agency within a virtual hand body illusion experiment is reduced. This response is dependent on visuomotor congruence and less on the morphological congruent arm [60].

The manipulation of visuomotor congruence, both on a spatial and spatiotemporal scale, may change the perception of space, as well as lead to an unconscious adaptation to visually modified movements in VR applications [61]. This unconscious motor response has been studied by Harvie et al., 2017, where altering the visual–kinaesthetic sensory information in VRi had an impact on the perceived movement and body position. These sensorimotor adaptations have been studied in patients with phantom limb pain syndrome [62], where the perception of a voluntary movement within a virtual reality system had an hypoalgesic effect.

### 3.4. Uncertainty about Outcomes

Similarly, sensorimotor uncertainty may have an impact on movement accuracy within a VRi environment. In a joint position precision discrimination task, the alteration of visual information produced a reduction in accuracy [63], which shows the importance of an adequate visuo-proprioceptive congruence in motor performance within VR.

Interestingly, this visuomotor adaptation capacity is preserved in patients with pain conditions such as fibromyalgia, despite alterations in their sensory perception and their poor ability to detect alterations in visual information provided by the virtual reality system [64]. Harvie et al. induced visual proprioceptive conflict during neck rotation, which affected the movement-evoked pain threshold in a VRi experiment [65].

Furthermore, an adequate multisensory congruence induces the sense of embodiment in a virtual body, which has a positive effect on motor performance. In a patient with an arm fracture, the embodiment-based immersive VR training program had positive results both in increasing the range of motion and in the functional capacity of the arm [66]. These effects were positively correlated with a greater sense of ownership and agency compared to non-VRi systems and conventional digital mobilisation. This shows that reducing the sensorimotor uncertainty within an VRi system could improve the motor capacity of patients with pain symptoms.

**Table 2:** Topic organisation considering the evidence for sensorimotor uncertainty from immersive virtual reality. Sensorimotor uncertainty could arise due to conflict or incongruence with motor or sensory functions, body or spatial representations, multisensory processing, and/or multisensory integration. For each of these conflicts, we consider evidence for motor decision-making stages related to pain experience.

Type of Sensorimotor Uncertainty	Immersive Virtual Reality Conflict	Correspondence with Motor Decision Making Stage
Uncertainty about sensory information	Visuotactile incongruence [40,41,43,67] Visuo-auditory incongruence [45,46,47] Visuo-vestibular incongruence [50,51]	Multisensory integration
Uncertainty about current state	Visuo-proprioceptive incongruence [42,52,68,69] Peripersonal space (immersion and presence) [29,58,70] Body illusions [54,55,56,71]	Body embodiment
Uncertainty about transition rule	Visuomotor incongruence [59,60,61,62,72] Virtual mirror therapy [73,74,75,76,77,78,79]	Motor performance
Uncertainty about outcome	Sensorimotor conflicts [39,65,80,81,82,83] Movement accuracy [63,64,66,84,85,86,87,88]	Reinforcement learning

Our system uses multisensory information to estimate surrounding features and to interact with objects. Visuotactile congruence has been studied to understand how our system is capable of estimating the weight of an object, within an illusion created using VRi [39]. This study shows that in case of a sensorimotor conflict with incongruent visuotactile stimuli, tactile cues have a stronger influence on perceived heaviness than visual cues. Furthermore, the interaction with virtual objects in an immersive environment influences the pain intensity [29]. These results show that interaction with objects increases the perception of presence, decreases pain intensity, and modulates threat perception compared to a passive virtual reality.

## 4. Discussion

Noise and uncertainty in our sensory and motor systems might have various impacts on task execution. This noise, while seemingly inherent at the neural level, appears to manifest behaviourally. Immersive virtual reality seems to provide an innovative non-pharmacological approach that could be effective for pain management. Several studies suggest its potential efficacy in managing nociceptive pain. Additionally, its possible role in treating neuropathic pain in conditions such as phantom limb pain, complex regional syndrome, or neuropathic pain in spinal cord injury patients has been explored. While the evidence hints at its promising efficacy, certain limitations should be taken into account.

The link between sense of ownership and motor behaviour has been reported in an experiment using non-virtual visual feedback, in which the muscle activity and movement speed decreased after a 150 ms delay in visual feedback in healthy patients [89]. This relationship has also been described in an experiment with immersive VR, in which a 200 ms delay in reaction times was observed when presented with incongruent feedback [85]. However, no effects have been reported on the level of delay in visual feedback in immersive VR, which may have an impact on motor performance. The alteration of not only temporal but also spatial visual feedback has also been studied in immersive VR, where it has been found that manipulating the amplitude of the virtual avatar’s movement through body ownership illusions may influence motor performance [83]. Lastly, an impact on motor performance has also been reported in an experiment comparing body illusions through virtual avatars in immersive VR featuring hands connected by arms or discontinuous hands. The findings of this study reflected that the participants’ motor performance was enhanced in the connected hand condition compared to the disconnected condition, without affecting the subject’s sense of agency [90]. Therefore, possible delays or alterations in sensory information need to be reported in more detail in experimental studies in order to understand what role it may play in sensorimotor uncertainty in motor behaviour.

Immersive virtual reality has been proposed as an innovative solution for the non-pharmacological management of people with pain, both acute and chronic [91]. The potential profiles that may benefit from a virtual reality-based intervention have been studied. Patients with both clinically and experimentally induced nociceptive pain show good results in decreasing the pain experienced [27]. 

Moreover, the potential role of VR in the treatment of neuropathic pain in various clinical conditions such as phantom limb pain, complex regional syndrome, or neuropathic pain in people with spinal cord injury (SCI) has been studied [92,93,94]. The current evidence provides promising results on the hypoalgesic effect of VR, although no firm conclusions can be drawn due to the limited quality of the studies conducted. Similarly, many of the studies that have explored VR interventions in neuropathic pain have used non-immersive VR devices. This is likely to have an impact on outcomes as pointed out by Donegan et al. [92], resulting in altered bodily perceptions that are thought to be associated with maladaptive structural and functional disturbances in the somatosensory cortex. These disturbances, which are present in many patients with neuropathic pain, may be an interesting therapeutic target in the VR approaches.

Several authors highlight the importance of introducing a perceptual experience of virtual embodiment to induce or facilitate neuronal plasticity processes in patients with neuropathic pain [92,95]. The manipulation of embodiment in pain patients by means of full-body avatar body illusions (BOIs) requires that sensorimotor uncertainty is minimised in the process of multisensory integration, as has been extensively studied in the rubber hand paradigm [96].

In patients with nociplastic pain associated with clinical conditions such as fibromyalgia or chronic migraine headaches, analgesic effects and improvements in function after interaction with VRi have also been reported. These authors recently hypothesised which mechanisms may have an impact on motor decision making in people with chronic musculoskeletal pain following VRi intervention [23].

In seeking to enhance clinical outcomes, as well as to better design and develop immersive VR software, it is necessary to deepen our knowledge of the mechanisms involved in VR-mediated hypoalgesia and changes in motor behaviour in people with pain.

### 4.1. Limitations of the Study

Several of the chosen studies might be subject to publication biases, and it is conceivable that unpublished studies with negative results might not have been included. The variety of VR devices and software used introduces variability that possibly influences the final outcomes. Moreover, patient-reported outcomes, like pain intensity or the sense of presence in VR, might inherently be subjective and differ from one individual to another. It is also noteworthy that some studies might have had small sample sizes, which potentially limits the generalizability of their findings. In terms of long-term effects and benefit retention, these have not been extensively examined yet. The precise neural mechanisms through which VR modulates pain perception largely remain a mystery. Lastly, different pain conditions might respond differently to VR interventions, and not all the studies have taken these nuances into account. It is also important to note that due to the nature of our review, a methodological quality assessment of the studies has not been conducted, so the results should be approached with caution.

### 4.2. Future Research Directions

A pivotal area of exploration lies in patient profiling for pain management. The determination of which specific pain patient profiles are most responsive to modifications in sensorimotor uncertainty is of the utmost importance. By accurately pinpointing these profiles, there lies an opportunity to craft targeted VRi treatments. In addition to this, there is a pronounced need for future studies to maintain meticulous documentation pertaining to the specifics of the VR interventions. This documentation should encompass not only detailed specifications of the devices used but also the nuanced characteristics of the software and any potential challenges that might be encountered during interventions.

A profound understanding of how the technical intricacies of VRi hardware influences sensorimotor uncertainty is crucial. This mandates a closer look into the contributions of different devices, ranging from head-mounted displays to sophisticated haptic feedback tools, all in relation to the patient’s overall experience and the outcomes of the treatment. Furthermore, given the inherent flexibility of VRi, it becomes imperative to scrutinise how alterations in software parameters, such as environment richness, multisensory integration, and body illusions, can impact sensorimotor uncertainty and, in turn, influence clinical outcomes.

While the current emphasis predominantly centres on VRi, drawing comparisons with non-immersive VR systems could yield instructive insights. Such comparative studies can shed light on the unique benefits and potential limitations inherent in each approach. Lastly, stepping beyond the realm of pain, there is a burgeoning interest in discerning how VRi can influence motor decision making, especially in the backdrop of other disorders, thereby potentially broadening its therapeutic application spectrum.

## 5. Conclusions

Immersive virtual reality alters sensorimotor uncertainty, increasing or decreasing it depending on the response required. It is possible to adjust different software features such as enriched environments, multisensory integration, body illusions, interaction with objects, and specific motor tasks. Similarly, technical specifications of VRi hardware, including head-mounted devices, controllers, or haptic devices, may influence sensorimotor uncertainty. Understanding the circumstances in which a person with pain is likely to face this uncertainty might help both VRi developers and clinicians to enhance the effects and diminish adverse effects.

## Figures and Tables

**Table 1:** Eligibility criteria of the literature search.

Inclusion Criteria	Exclusion Criteria
Randomised clinical trials, secondary analysis of randomised clinical trials, and pilot randomised clinical trials	Studies that included other types of non-immersive VR intervention, and/or non-virtual intervention(s) Studies about children
Immersive virtual reality interventions compared to [i] no intervention; [ii] sham control; [iii] usual care control; or [iv] active control.	Full text not available Protocol for randomised clinical trials
Published in peer reviewed journal or conference proceedings	
Published since year 2018	
Written in English language	

## Data Availability

Not applicable.

## Appendix A

**Table:**

Date and Author	Type of Sensorimotor Uncertainty	Immersive VR Conflict	Correspondence with Motor Decision Making Stage	Main Findings
Rubo et al., 2019 [40]	Uncertainty about Sensory information	Visuo-tactile incongruence	Multisensory inte-gration	Action-oriented, unconscious body schema relies more heavily on tactile information compared to more explicit aspects of body ownership
Bekrater-Bodmann et al., 2014 [41]	Uncertainty about Sensory information	Visuo-tactile incongruence	Multisensory inte-gration	The temporal limits of the induction of limb ownership related to multisensory body-related input, suggesting their involvement in the processing of bodily awareness through the integration of visual and tactile events
Sano et al., 2016 [43]	Uncertainty about Sensory information	Visuo-tactile incongruence	Multisensory inte-gration	The tactile feedback improves the immediate pain intensity through rehabilitation using our virtual reality system
Mattsson et al., 2022 [67]	Uncertainty about Sensory information	Visuo-tactile incongruence	Multisensory inte-gration	Visuo-tactile temporal correlations have a stronger influence on body ownership than visuo-vestibular correlations and that ownership boosts self-motion perception
Naef et al., 2022 [45]	Uncertainty about Sensory information	Visuo-auditory incongruence	Multisensory inte-gration	The use of audiovisual VR stimulation is more effective at inducing a relaxation response compared to no artificial sensory inputs, or the independent inputs.
Khan et al., 2020 [46]	Uncertainty about Sensory information	Visuo-auditory incongruence	Multisensory inte-gration	Avatar’s movements can be used to influence a person’s own motion, but should include relevant auditory cues congruent with the movement to ensure a suitable level of entrainment is achieved.
Liu et al., 2020 [47]	Uncertainty about Sensory information	Visuo-auditory incongruence	Multisensory inte-gration	Depth localization of a visual object in virtual reality can be altered by a spatially incongruent sound, and provide a potential approach that we can adopt a spatially incongruent sound as a cue to reduce the depth compression in VR
Ng et al., 2020 [50]	Uncertainty about Sensory information	Visuo-vestibular incongruence	Multisensory inte-gration	When users are placed under a visual-vestibular synchronised condition, their subjective miserable score of cybersickness decreased while their comfort level of the overall experience increased
Y So et al., 2001 [51]	Uncertainty about Sensory information	Visuo-vestibular incongruence	Multisensory inte-gration	The nausea and vection ratings increased significantly with speeds increasing from 3 m/s to 10 m/s. At speeds exceeding 10 m/s, the ratings stabilized. Navigation speeds were found to significantly affect the onset times of vection and nausea but did not affect their rates of increase with duration of exposure. Navigation speed had a significant influence on only the oculomotor subscore of SSQ
Ichinose et al., 2017 [42]	Uncertainty about Current state	Visuo-proprioceptive incongruence	Body embodiment	The analgesic effect of visual feedback during phantom limb movement is significantly improved by applying somatosensory feedback to the cheek on the affected side
Fossataro et al., 2020 [52]	Uncertainty about Current state	Visuo-proprioceptive incongruence	Body embodiment	When vision and proprioception are congruent (i.e., real and virtual hand coincide), a space-dependent modulation of the visual enhancement of touch (VET) effect occurs (with faster responses when visual stimuli are near to than far from the stimulated hand). Contrarily, when vision and proprioception are incongruent (i.e., a discrepancy between real and virtual hand is present), a comparable VET effect is observed when visual stimuli occur near to the real hand and when they occur far from it, but close to the virtual hand
Alemanno et al., 2019 [68]	Uncertainty about Current state	Visuo-proprioceptive incongruence	Body embodiment	Teaching patients to execute correct movements with the painful body parts to regain a correct body image, based on the augmented multisensory feedback (auditory, visual) provided by the VR shows significant reductions in all pain rating scale scores (*p* < 0.05); significant improvements of QoL in the domains of physical functioning, physical role functioning, bodily pain, vitality, and social role functioning; improvements in cognitive functions (*p* < 0.05); improvements in functional scales (*p* < 0.05) and mood (*p* = 0.04).
Limanowski et al., 2020 [69]	Uncertainty about Current state	Visuo-proprioceptive incongruence	Body embodiment	Endogenous attention can balance the gain of visual versus proprioceptive brain areas, thus contextualizing their influence on multisensory areas representing the body for action
Cooper et al., 2018 [58]	Uncertainty about Current state	Peripersonal space (immersion and presence)	Body embodiment	Participants performed best and felt an increased sense of immersion and involvement, collectively referred to as ’presence’, when substitute multimodal sensory feedback was provided. Significant main effects of audio and tactile cues on task performance and on participants’ subjective ratings were found. A significant negative relationship was found between the objective (overall completion times) and subjective (ratings of presence) performance measures
Scandola et al., 2020 [70]	Uncertainty about Current state	Peripersonal space (immersion and presence)	Body embodiment	The presence of motor feedback was necessary for the recovery of Peripersonal space (PPS) representation, both when the motor feedback was congruent and when it was incongruent with the visual feedback. In contrast, visuo-motor incongruence led to an inhibition of PPS representation in the control group
Hoffman et al., 2021 [29]	Uncertainty about Current state	Peripersonal space (immersion and presence)	Body embodiment	Interacting with virtual objects via embodied avatar hands (i.e., avatar VR)significantly increased the participant’s illusion of “being there” in the virtual world, increased VR analgesia, andincreased fun during the pain stimulus.
Matamala-Gomez M et al., 2020 [54]	Uncertainty about Current state	Body illusions	Body embodiment	Positive relationship between the level of ownership over the distorted and reddened-distorted virtual arms with the level of pain/discomfort, but not in the normal control arm
Matamala-Gomez M et al., 2021 [55]	Uncertainty about Current state	Body illusions	Body embodiment	Patients with chronic pain can achieve levels of ownership and agency over a virtual arm similar to healthy participants. This multisensory interventions by manipulating the body representation throughVR can modulate pain perception
Martini et al., 2013 [56]	Uncertainty about Current state	Body illusions	Body embodiment	Influence of skin color on pain perception. This top-down modulation of pain through visual input suggests a potential use of embodied virtual bodies for pain therapy
Pyasik et al., 2020 [72]	Uncertainty about Current state	Body illusions	Body embodiment	Subjective ownership of the own hand (OH) was stronger than of the fake hand (FH) in congruent location after synchronous stimulation. It was also present after asynchronous stimulation, being stronger when the virtual OH was subjectively more similar to the real hand. The results suggest that the detailed appearance of the body might act as an additional component in the construction of body ownership.
D’Angelo M et al., 2018 [59]	Uncertainty about Transition Rule	Visuo-motor incongruence	Motor performance	Body schema and peripersonal space are affected by the dynamic between intentional body movements and expected consequences in space
Brugada-Ramentol et al., 2019 [60]	Uncertainty about Transition Rule	Visuo-motor incongruence	Motor performance	Congruent active control enhanced and maintained the reported sense of ownership. Incongruent active control, achieved by adding noise to the trajectory of the movement, decreased both reported sense of agency and ownership.
Kokkinara E et al., 2015 [61]	Uncertainty about Transition Rule	Visuo-motor incongruence	Motor performance	Spatiotemporal manipulation of 2 and 4 times faster can significantly change participants’ proprioceptive judgments of a virtual object’s size and the agency of the movements.
Osumi M et al., 2017 [62]	Uncertainty about Transition Rule	Visuo-motor incongruence	Motor performance	Using a bimanual coordination task correlated with alleviation of phantom limb pain
Buetler K et al., 2022 [73]	Uncertainty about Transition Rule	Visuo-motor incongruence	Motor performance	The reported illusion strength was associated with and faster movement initiations, indicating that participants may have physically mirrored and compensated for the body characteristics of the avatar
Barton et al., 2014 [74]	Uncertainty about Transition Rule	Virtual mirror therapy	Motor performance	Dynamic morphing using Virtual Mirror Box resulted in a compromise between mirrored movement of the intact side and gait events of the virtual limbs matched with physical events of the impaired side
Hsu et al., 2022 [75]	Uncertainty about Transition Rule	Virtual mirror therapy	Motor performance	Virtual reality Mirror Therapy had the same effects in restoring the upper extremity motor function as actual Mirror Therapy
Murray et al., 2007 [78]	Uncertainty about Transition Rule	Virtual mirror therapy	Motor performance	All participants reported the transferal of sensations into the muscles and joints of the phantom limb, and a decrease in phantom pain during at least one of the sessions
Weber et al., 2019 [79]	Uncertainty about Transition Rule	Virtual mirror therapy	Motor performance	Motor outcomes did not achieve statistical significance using Immersive VR mirror therapy
Mazzola et al., 2020 [80]	Uncertainty about Transition Rule	Virtual mirror therapy	Motor performance	There was no significant difference in time between the mirrored and virtual-normal conditions
Naylor et al., 2021 [39]	Uncertainty about Outcome	Sensorimotor conflicts	Reinforcement learning	Expectations derived from tactile material cues exert a more substantial influence on heaviness perception, compared to visual material cues
Harvie et al., 2015 [65]	Uncertainty about Outcome	Sensorimotor conflicts	Reinforcement learning	Visual-proprioceptive information modulated the threshold for movement-evoked pain
Berger et al., 2022 [81]	Uncertainty about Outcome	Sensorimotor conflicts	Reinforcement learning	Positive correlation between the extent of the outward drift of the participants’ arm and the perceived reachability of distal objects
Gordon et al., 2019 [82]	Uncertainty about Outcome	Sensorimotor conflicts	Reinforcement learning	Effects on pain threshold were present for type of visuo-tactile stimulation but not type of movement
Bourdin et al., 2019 [83]	Uncertainty about Outcome	Sensorimotor conflicts	Reinforcement learning	Altered visual feedback through body ownership illusions can influence motor performance
Spitzley et al., 2022 [63]	Uncertainty about Outcome	Movement accuracy	Reinforcement learning	When available, vision was relied upon more heavily than proprioception for task completion
Dagenais et al., 2021 [64]	Uncertainty about Outcome	Movement accuracy	Reinforcement learning	Altering visual feedback did not influence pain during a reaching task, and both groups adapted similarly to it
Matamala-Gómez et al., 2022 [66]	Uncertainty about Outcome	Movement accuracy	Reinforcement learning	Functional recovery was correlated with the ownership and agency scores over the virtual arm. Larger range of joint movements and lower disability
Odermatt et al., 2021 [85]	Uncertainty about Outcome	Movement accuracy	Reinforcement learning	Congruency of information create subjective body ownership and is associated with faster reaction times
Harvie et al., 2017 [86]	Uncertainty about Outcome	Movement accuracy	Reinforcement learning	Altered visual feedback caused a kinaesthetic drift in the direction of the visually suggested movement
Yamada et al., 2021 [87]	Uncertainty about Outcome	Movement accuracy	Reinforcement learning	Better performance, specifically greater accuracy and lower one-dimensional bias in the anteroposterior direction when adopting an external attentional focus
Aoyagi et al., 2021 [88]	Uncertainty about Outcome	Movement accuracy	Reinforcement learning	Sense of agency can be enhanced by modifying feedback to motor tasks according to the goal of the task, even when visual feedback is discrepant from the actual body movements
